# Expression of Concern: Stripes and loss of color in ball pythons (*Python regius*) are associated with variants affecting endothelin signaling

**DOI:** 10.1093/g3journal/jkad182

**Published:** 2023-08-23

This is an expression of concern about *G3 Genes|Genomes|Genetics*, Volume 13, Issue 7, July 2023, jkad063, https://doi.org/10.1093/g3journal/jkad063.

Shortly after article publication, the authors of this article alerted the Editorial Office to the use of erroneous gene names for endothelin receptor genes. The correct annotation for the gene associated with color phenotypes (accession OP589186) is EDNRB2, and the correct annotation for the gene not associated with color phenotypes (accession OQ412607) is EDNRB1.

These corrected annotations reflect orthology relationships with B-type endothelin receptor genes in other vertebrates. An implication of this correction is that ball pythons are similar to birds in that EDNRB2 is dispensable for viability and is associated with color variants. EDNRB1, by contrast, may not be dispensable for viability in ball pythons, given that no variants in this gene were identified. No other results or conclusions appear to be impacted by these errors.

While the authors correct the annotations within their article, readers are urged to examine this study with care.

