# Supplementary figures and images for: Stripes and loss of color in ball pythons (Python regius) are associated with variants affecting endothelin signaling
Source: G3 (Bethesda). 2023 May 16;13(7):jkad063. doi: 10.1093/g3journal/jkad063 (PMC10320763; doi:10.1093/g3journal/jkad063)

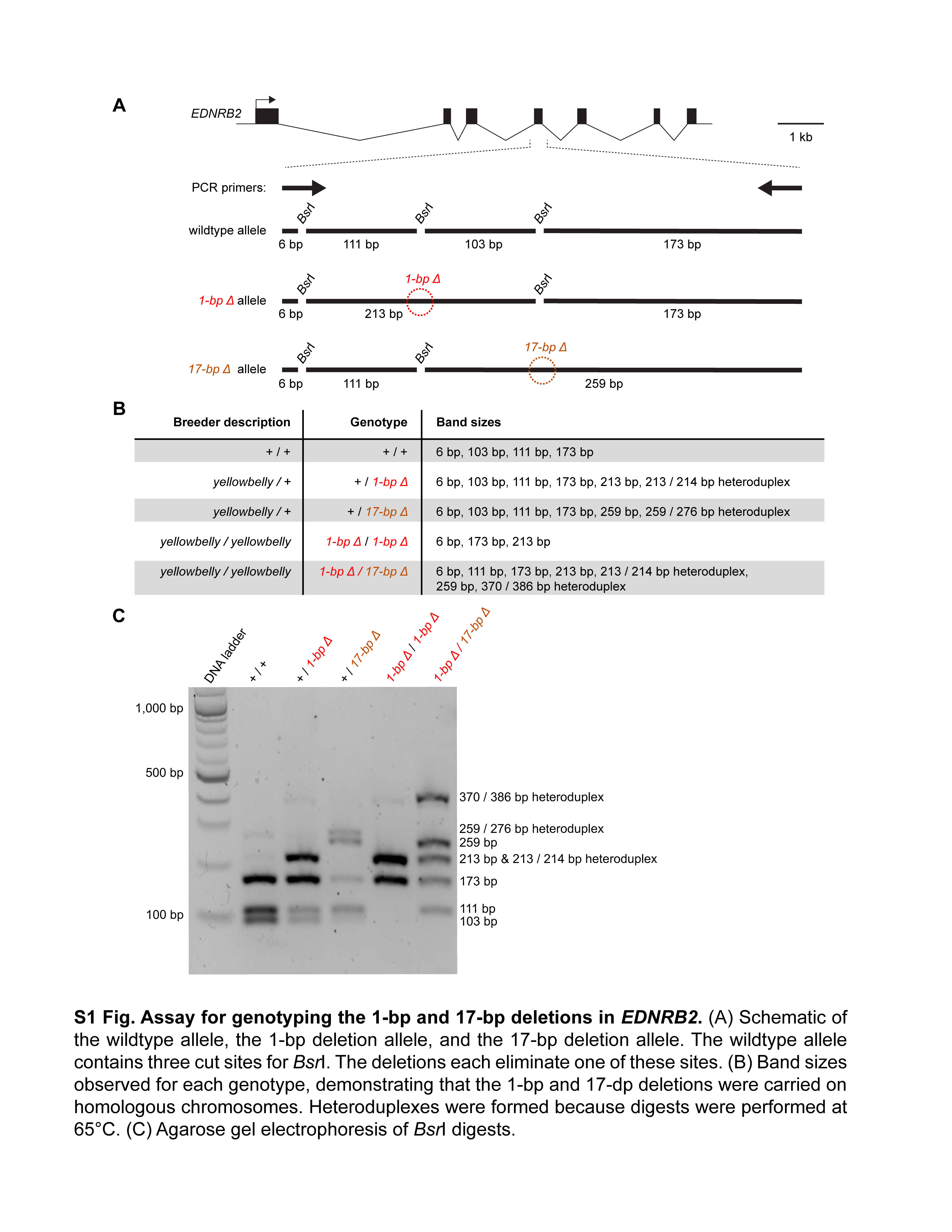

Supplement: jkad063_Supplementary_Data [file jkad063_supplementary_data.zip › jkad063_corrected_S1 Fig.tif]

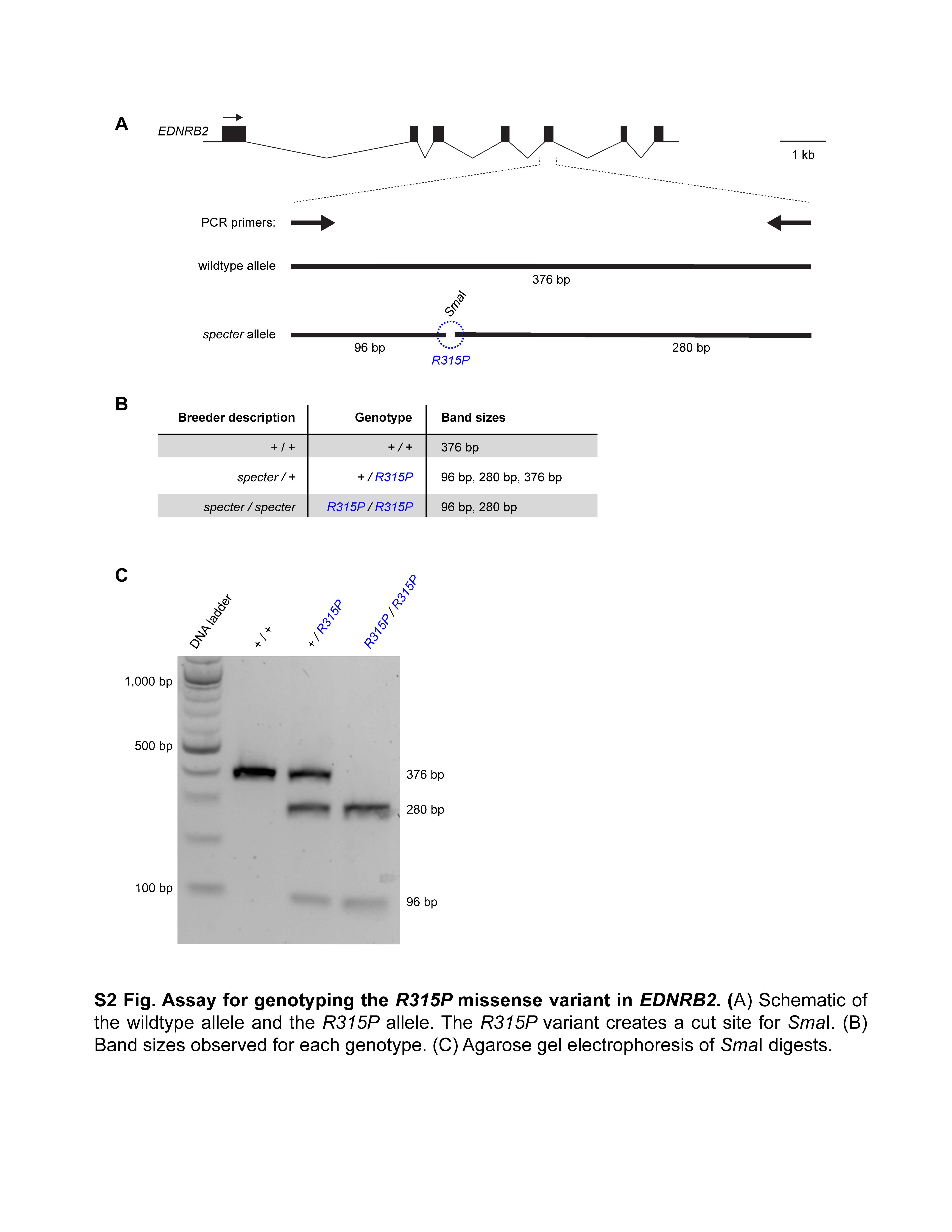

Supplement: jkad063_Supplementary_Data [file jkad063_supplementary_data.zip › jkad063_corrected_S2 Fig.tif]

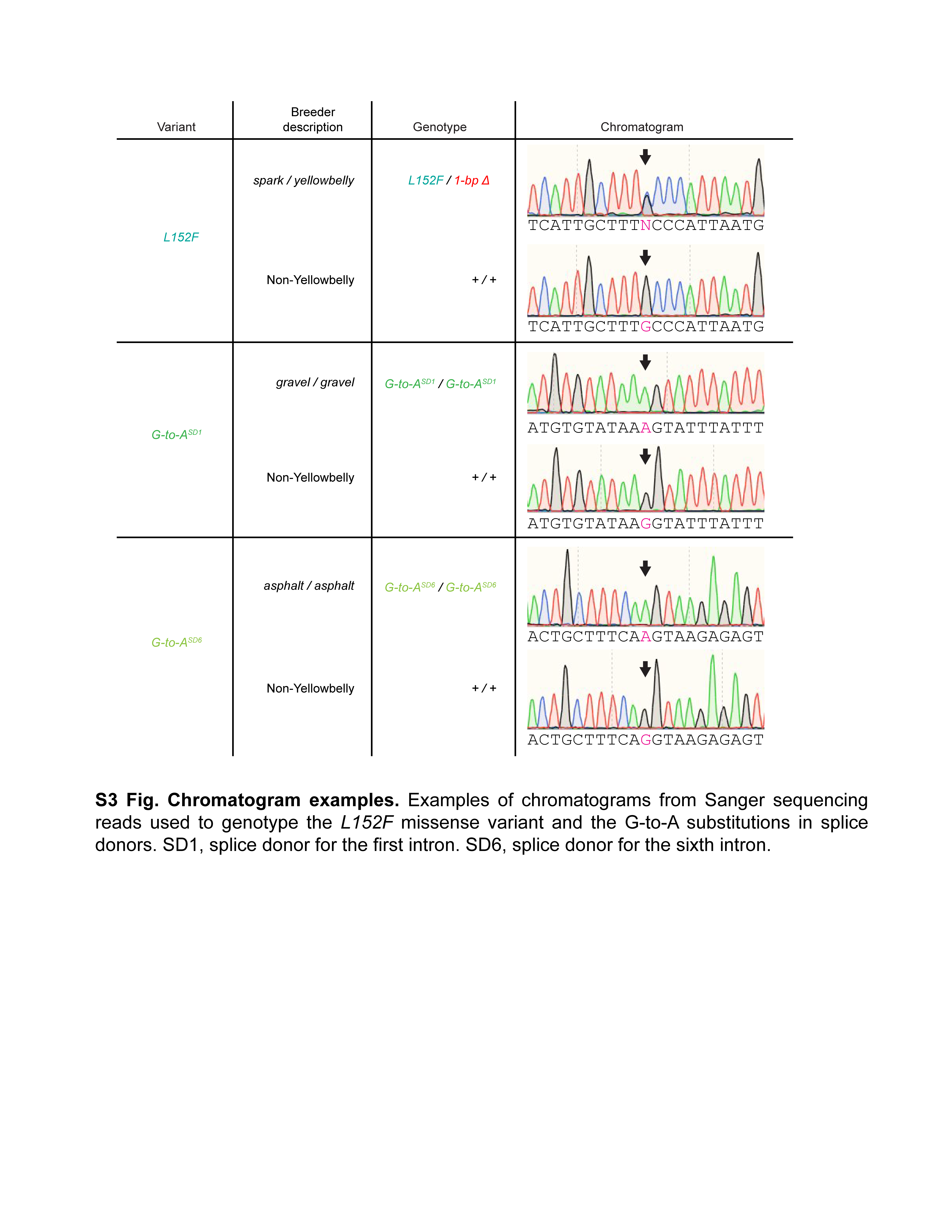

Supplement: jkad063_Supplementary_Data [file jkad063_supplementary_data.zip › jkad063_corrected_S3 Fig.tif]
